# The Diversified Impacts of Urban Morphology on Land Surface Temperature among Urban Functional Zones

**DOI:** 10.3390/ijerph17249578

**Published:** 2020-12-21

**Authors:** Sihang Gao, Qingming Zhan, Chen Yang, Huimin Liu

**Affiliations:** 1 School of Urban Design, Wuhan University, Wuhan 430072, China; sihanggao@whu.edu.cn; 2 Collaborative Innovation Centre of Geospatial Technology, 129 Luoyu Road, Wuhan 430079, China; 3 College of Urban and Environmental Sciences, Peking University, Beijing 100871, China; cyangcues@stu.pku.edu.cn; 4 Institute of Space and Earth Information Science, The Chinese University of Hong Kong, Shatin, NT, Hong Kong, China; hmliu@cuhk.edu.hk

**Keywords:** land surface temperature, urban functional zones, geographically weighted regression, random forest regression, urban morphology

## Abstract

Local warming induced by rapid urbanization has been threatening residents’ health, raising significant concerns among urban planners. Local climate zone (LCZ), a widely accepted approach to reclassify the urban area, which is helpful to propose planning strategies for mitigating local warming, has been well documented in recent years. Based on the LCZ framework, many scholars have carried out diversified extensions in urban zoning research in recent years, in which urban functional zone (UFZ) is a typical perspective because it directly takes into account the impacts of human activities. UFZs, widely used in urban planning and management, were chosen as the basic unit of this study to explore the spatial heterogeneity in the relationship between landscape composition, urban morphology, urban functions, and land surface temperature (LST). Global regression including ordinary least square regression (OLS) and random forest regression (RF) were used to model the landscape-LST correlations to screen indicators to participate in following spatial regression. The spatial regression including semi-parametric geographically weighted regression (SGWR) and multiscale geographically weighted regression (MGWR) were applied to investigate the spatial heterogeneity in landscape-LST among different types of UFZ and within each UFZ. Urban two-dimensional (2D) morphology indicators including building density (BD); three-dimensional (3D) morphology indicators including building height (BH), building volume density (BVD), and sky view factor (SVF); and other indicators including albedo and normalized difference vegetation index (NDVI) and impervious surface fraction (ISF) were used as potential landscape drivers for LST. The results show significant spatial heterogeneity in the Landscape-LST relationship across UFZs, but the spatial heterogeneity is not obvious within specific UFZs. The significant impact of urban morphology on LST was observed in six types of UFZs representing urban built up areas including Residential (R), Urban village (UV), Administration and Public Services (APS), Commercial and Business Facilities (CBF), Industrial and Manufacturing (IM), and Logistics and Warehouse (LW). Specifically, a significant correlation between urban 3D morphology indicators and LST in CBF was discovered. Based on the results, we propose different planning strategies to settle the local warming problems for each UFZ. In general, this research reveals UFZs to be an appropriate operational scale for analyzing LST on an urban scale.

## 1. Introduction

Changes in urban function and urban form have collectively modified the urban heat balance, resulting in local warming at the intra-urban scale, which has raised significant concerns among urban researchers [1,2]. The transformation from natural surfaces to a built up environment has led to changes in ecosystem services and surface physical properties, for example, altered the surface albedo, heat storage capacity, and thermal conductivity properties [3,4,5,6]. Besides, accompanying alteration in the surface three-dimensional morphology impedes ventilation [7,8]. These combined effects increase the difficulties in heat exchange between urban areas and the surrounding environment [9]. Land surface temperature (LST) images derived from satellite sensors are widely used to represent the urban thermal environment, which are more convenient and intuitive because they provide full spatial coverage compared to air temperatures obtained from weather stations [10,11]. The correlation between LST and urban characteristics such as impervious surface fraction, vegetation indexes, and building indexes has been well documented at the intra-urban scale, facilitating the translation of interaction knowledge between the physical form and the climatic context into practical planning applications [12,13,14,15]. As a result, this study aims to convey further knowledge about how LST is impacted by potential landscape drivers [16].

Conventional urban LST researches have followed the macro-scale “urban-rural” dichotomy, which is insufficient in capturing local diversity [17], while planning professionals demand more straightforward guidelines at a local scale [18]. Neglecting spatial heterogeneity in the landscape-LST relationship can result in an underestimation of LST and its effects, thus exposing susceptible populations to an actual higher illness risk [19,20]. Besides, “urban-rural” dichotomy studies focus more on the LST mechanism’s differences between built up and non-built-up areas, so it is difficult for urban planners to translate this abstract knowledge into practical planning control means [21]. In fact, urban planners need more direct maps and models to guide the arrangement of landscape composition, urban configuration, and urban morphology to address urban climatic issues. Thus, the idea of urban zoning is introduced to explore these diversified impacts in addition to the conventional “urban-rural” dichotomy of LST differences [22]. One of the extensive discussions is the local climate zone (LCZ), which divides urban areas through different composition, configuration, and morphology of the land surface, resulting in homogeneous units with uniform land cover, structure, materials, and human activities [23,24,25,26].

In recent years, many researchers have proposed a number of extensions to the conventional LCZ model, one of which is widely used is urban functional zone (UFZ), due to its close connection to planning practice. For urban planners, UFZ is an appropriate framework as it facilitates the development of differentiated planning control measures that can be linked to micro-scale detailed planning [27,28]. UFZs divide urban space according to the differences in socioeconomic activities, which reflect differences in human behavior patterns, intensity of activity, and population density [29]. In fact, an increasing number of researchers in recent years have introduced urban functional zoning into their studies [24,30,31,32]. The landscape-LST relationship among UFZs is discussed at two levels: (1) each type of UFZ has different human activities and anthropogenic heat, thus spatial landscape indicators have diversified impacts on LST among UFZs; (2) in the same type of UFZ, each type of landscape indicators’ impact process on LST may not occur at the same spatial scale, thus resulting in different impacts of spatial landscape indicators on LST at different locations. For the research of the spatial heterogeneity in the Landscape-LST relationship among UFZs, the different UFZs need to be regarded as a whole to reflect the diversified impact at the macro-scale and the spatial heterogeneity in the Landscape-LST relationship in specific UFZ should be discussed by a separate model for each UFZ [24].

This paper selects conventional landscape composition indicators and urban form indicators for regressions to investigate the spatial heterogeneity in their correlations with LST both among UFZs and within each UFZ. Different UFZs have different building forms, landscape compositions, and socioeconomic activities with their own heat production, heat absorption, and heat exchange characteristics, which have diversified impacts on the local urban thermal environment, leading to local LST variability [33]. In previous urban LST researches, the impervious surface fraction and the vegetation cover ratio showed a high correlation with LST. Therefore, controlling the built up area proportion and increasing ecological land use have been seen as effective ways to mitigate the local warming effect [34]. Nevertheless, the urban vegetation cover proportion is often limited by socioeconomic development. This is why more and more researchers have been focusing on the urban three-dimensional morphology in recent years [26,30,34,35]. On the one hand, urban buildings can alter the surface reflection and absorption of solar radiation, as well as the urban surface roughness, and thus, can affect the surface ventilation and heat exchange. On the other hand, controlling the urban building form is the most important tool for planners to develop planning strategies to influence the urban environment in built up areas. In fact, due to the different urban functions, the construction standards across different UFZs vary considerably, leading to significant differences in the building form of different UFZs and different impacts on the urban thermal environment. In existing studies, building form indicators are divided into two-dimensional (2D) and three-dimensional (3D) buildings form indicators. The 2D buildings form indicators include building density (BD) and the 3D buildings form indicators include building height (BH) [36], building volume density (BVD), and sky view factor (SVF) [30,37,38,39]. Both of them are considered simultaneously in this paper and analyzed separately.

Since conventional multiple linear regression cannot well explain the spatial heterogeneity in the Landscape-LST relationship, two improved geographically weighted regression (GWR) models including semi-parametric geographically weighted regression (SGWR) models and multiscale geographically weighted regression (MGWR), were introduced in the research within each type of UFZ [40,41]. Based on the conventional ordinary least square (OLS) regression, the GWR model incorporates a spatial weight matrix to represent this spatial autocorrelation, while the predictions are calibrated according to position and weighting [33,40]. GWR is considered an appropriate framework for urban problems because it takes into account the non-stationarity of spatial variables [21]. Urban planners are able to differentiate their planning strategies based on the spatial distribution of slope coefficients for each indicator, bridging the gap between urban climate research and urban planning applications [42,43]. In contrast to the classical GWR model, which assumes that all phenomena occur at the same spatial scale, the MGWR and SGWR models allow the different phenomena to have different spatial scales. In fact, the spatial scales at which different types of landscape indicators function in urban thermal environment studies are likely to be different [44,45,46]. Besides, on account of the prevalence overfitting in GWR, random forest (RF) regression and OLS serve as pre-regressions to compare the different impacts of landscape indicators on LST across UFZs. The regression results will also be used as a reference for the GWR model to exclude landscape indicators that contribute weakly to the LST in UFZ. The RF regression is a machine learning model that is generally considered effective in reducing overfitting in high-dimensional data processing and is able to tap into the potential impact patterns of the phenomenon [47,48].

Based on the above framework, this research aimed to: (1) discover the differences in LST and landscapes among UFZs, (2) explore the different urban morphology’s impacts on LST both among UFZs and within each UFZ, (3) examine whether the impact process of different landscape indicators on LST operate in different scales, (4) provide knowledge for urban planning to mitigate the local warming effect based on the UFZs unit.

## 2. Study Area and Data Sets

### 2.1. Study Area

**Figure 1:**
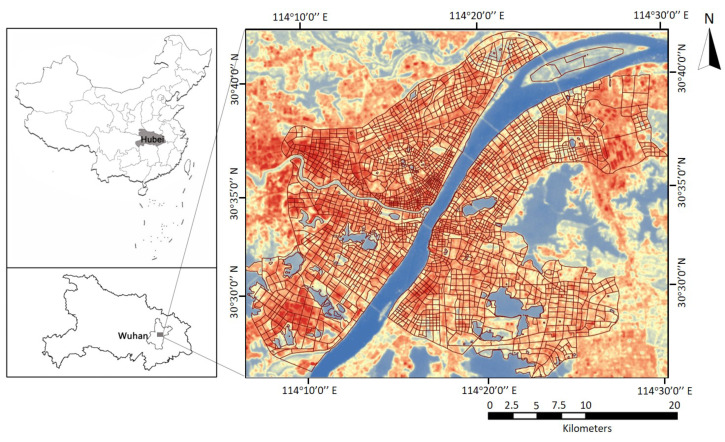
The study area represented by the land surface temperature image retrieved by Landsat 8 image (RGB) acquired on 16 August 2013.

Wuhan, a megacity and important industrial city located in central China, was selected as a case study to investigate the urban thermal dynamics affected by urban morphology and urban function in high density cities. Wuhan ranks as the fifth largest city in China, with a population of over 10 million. The total built up area of the city is about 888 km^2^ in 2013 and it reached 1999 km^2^ in 2018, among which industrial land accounts for 14.5%. Considered one of the four major furnaces in China, rapid urbanization resulted in severe local warming effects in Wuhan. The urban thermal dynamic exploration of Wuhan will contribute to the urban function organization and the local warming mitigation policies formulation for mega-cities. The study area was also sufficient to illustrate the heterogeneity of urban morphology and urban function with diversified vegetation areas, industrial areas, and build-up areas and eliminated the interference caused by the water bodies (as shown in Figure 1).

### 2.2. Data Sets

**Figure 2:**
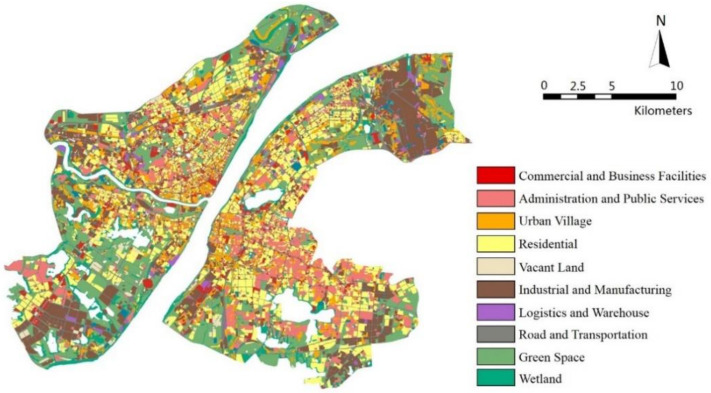
The urban functional zones (UFZs) map acquired by land inventory in 2013.

Multi-source data, including Landsat 8 images, building survey data, and UFZs survey data were utilized in this study. Images collected from the Landsat-8 OLI (Operational Land Imager) and TIRS (Thermal Infrared Sensor) were selected on account of the high resolution of 30 m, which is able to facilitate the exploration of street-scale surface temperature mechanisms. According to historical data, July and August represent the hottest months over the years in Wuhan, so cloud-free Landsat-8 images collected from 16 August 2013 were selected. The land surface temperature image and some of the landscape indicators were retrieved by the Landsat-8 images. Besides, building survey data and the urban functional zones map of 2013 provided by the Land Resources and Planning Information Center of Wuhan were utilized to retrieve urban morphology indicators. Both types of data were manually corrected by satellite imagery in August 2013 from Google Earth to determine that they matched the actual conditions. In general, both functional maps and buildings data were more than 80% accurate. Among them, urban functional zones were reclassified into 10 categories to investigate the relationship between LST and urban function, which were Administration and Public Services (APS), Commercial and Business Facilities (CBF), Green Space (GS), Industrial and Manufacturing (IM), Logistics and Warehouse (LW), Residential (R), Road and Transportation (RT), Urban Village (UV), Vacant Land (VL), and Wetland (W) (as is shown in Figure 2). UFZs are divided according to national regulation and planners’ habits, with reference to the way they have been divided in previous studies. The smaller UFZ units were combined in order to reduce interference from chance errors due to land size.

## 3. Methodology

**Figure 3:**
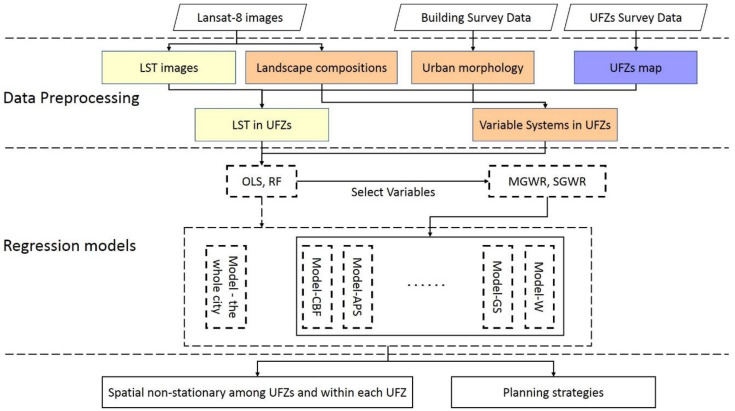
The methodological framework used in this study.

The methodological framework was adopted to explore the spatial heterogeneity in the Landscape-LST relationship among different UFZs and within each UFZ, as shown in Figure 3 below. It is divided into the following four steps: (1) retrieve the LSTs from Landsat 8 thermal infrared sensor (TIRS) images; (2) obtain landscape composition and urban morphology indicators, respectively, from Landsat 8 images and building survey data; (3) model the associations between LST and landscape indicators through random forest regression to investigate the diversified impact among different UFZs; (4) analyze the variation of the Landscape-LST spatial non-stationary associations modelled via SGWR and MGWR.

### 3.1. The Retrieval of LST

In this paper, the selected Landsat 8 TIR scene was used to retrieve LST through the classic radiative transfer equation (RTE) [49,50]. It is a kind of physical single-window method, which uses the radiation transfer model and the synchronous atmospheric profile put forward by Barsi in 2003 [51] for atmospheric correction. Yu et al. compared the RTE method, the split-window methods, and the mono-window algorithms by using the measured data of four SURFRAD (surface radiation data) sites [50]. The results showed that the RTE method had the highest accuracy, with the Root Mean Square Error (RMSE) less than 1 K. A simplified radiative transfer equation can express the apparent radiance received by a sensor,

(1) $$L_{\lambda }=\left[\varepsilon B\left(T_{S}\right)+\left(1-\varepsilon \right)L↓\right]\tau +L↑,$$

where ε is the surface specific emissivity, $T_{S}$ is the actual surface temperature (K), $B\left(T_{S}\right)$ is the black body thermal radiance, and τ is the permeability of atmosphere in the thermal infrared band.

According to the Planck formula and sensor parameters of Landsat 8 TIRS, it can be translated into

(2) $$T_{S}=\frac{C_{1}}{\lambda \ln(\frac{C_{2}}{\lambda^{5}\left[\frac{B\left(T_{S}\right)-L↑-\tau \left(1-\varepsilon \right)L↓}{\tau \varepsilon }\right]}+1)},$$

where λ is the effective band wavelength, C1 = 14,387.7 μm, C2 = 1.19104 × 10^8^ W·μm^4^·m^−2^·sr^−1^. The atmospheric correction parameter calculator put forward by Barsi (2003) provided by National Aeronautics and Space Administration (NASA) was used to calculate the downwelling and upwelling atmospheric radiance [51].

### 3.2. The Selection of Landscape Indicators

Seven landscape indicators were selected by the LST dynamic and urban planning regulation. The landscape composition indicators derived from Landsat images cover NDVI and surface albedo, both of which were retrieved after atmospheric correction [33,52,53,54]. The impervious surface fraction (ISF) are derived from the open-source datasets (http://data.ess.tsinghua.edu.cn/) provided by Gong et al. [55]. In this data set, the exclusion/inclusion algorithm was adopted to obtain the impervious surface thresholds for each year by using the NDVI, MNDWI, and SWIR maps obtained from Landsat images. After the correction of NTL data and the temporal consistency check algorithm to reduce the time non-stationary interference, the overall accuracy of IS extractions is claimed to be higher than 93%. The urban morphology indicators calculated from building survey data contain BD, BH, BVD, and SVF [30,34,56]. The description and data range of the selected landscape composition and urban morphology indicators are presented in Table 1. All of these indicators were examined in terms of the multicollinearity using the variance inflation factor (VIF); those passing a tolerable multicollinearity (VIF < 10) were selected referring to previous research in order to mitigate the impact of collinearity on the estimation precision of the slope coefficient [33,40,42].

### 3.3. Random Forest Regression Model

The Random Forest Algorithm is a widely used machine learning algorithm for element classification and regression [57]. The decision tree is the basic unit of the random forest algorithm, which is an integrated algorithm that aggregates a large number of generated “trees” into a single prediction. In the random forest algorithm, each tree is involved in the decision making, while training data are randomly selected for modeling to reduce the error. The data are called out-of-bag and the final output of the model is the average of all the trees. The RF algorithm determines the importance of each predictor variable by assessing the increase in prediction error when the OOB of that variable changes while the other variables remain constant [58,59]. The advantage of the RF algorithm is that it is highly robust in terms of predicting the noise in it, while reducing overfitting of the model [47].

### 3.4. Geographic Weighted Regression Models

Geographic Weighted Regression was selected for quantifying the spatial non-stationarity of the associations between LST and landscape indicators, while OLS was adapted as pre-regression to identify the prominence and multicollinearity of selected indicators to reduce unexpected error in GWR [19]. The spatial distribution of the local coefficients estimated by GWR and each indicator’s local slope coefficients were displayed in images. Specifically, OLS is a widely employed global regression model that matches by minimizing the error square sum and finding the best function for the data. It can be expressed as

(3) $$y=\beta_{0}+\sum_{k}\beta X_{k}+e,$$

where y denotes the dependent variable, $X_{k}$ represents the *k*th independent variables, $\beta_{0}$ is the intercept value, *k* represents the number of independent variables, β denotes the slope coefficient of each independent variables, and *e* is the random error.

The GWR model is an extension of the OLS model, which takes into account the spatial heterogeneity [42]. In GWR, a local regression model is fitted at each data point (location) using distance-weighted subsamples defined by a bandwidth [56,60]. Specifically, GWR models the associations through:

(4) $$y_{i}=\beta_{0}\left(u_{i},v_{i}\right)+\sum_{k}\beta_{k}\left(u_{i},v_{i}\right)x_{ik}+\varepsilon_{i},$$

where $u_{i}$ and $v_{i}$ are the coordinates for each location *i*, $\beta_{0}\left(u_{i},v_{i}\right)$ is the intercept for location *i*, and $\beta_{k}\left(u_{i},v_{i}\right)$ is the local parameters estimate for independent variable $x_{k}$ at location *I*, by which the GWR extends the global regression model by adding the geographical location parameter to generate the local coefficients to account for spatial non-stationarity. The weight matrix is used to weight the observations differently, while the parameters are solved by the following matrix form:

(5) $$\beta_{0}\left(u_{i},v_{i}\right)={\left(X^{T}W\left(u_{i},v_{i}\right)X\right)}^{-1}X^{T}W\left(u_{i},v_{i}\right)Y,$$

where $W\left(u_{i},v_{i}\right)$ denotes a matrix of which its diagonal elements refer to the geographical weighting of the observation data considered for observation *i*. In this study, the fixed Gaussian kernel is utilized as the weighting regime to identify the local extents for model fitting, which continuously and gradually decreases from the center of the kernel but never reaches zero.

(6) $$w_{ij}=e^{-\frac{d_{ij}^{2}}{\theta^{2}}},$$

where *θ* is called the bandwidth of the kernel function and *d_ij_* represents the Euclidean distance between point *i* and *j*. According to the Gaussian curve, the $w_{ij}$ value is gradually decreasing as the distance *d_ij_* is increasing. The weight value will be set to zero if the distance is greater than the basil width of the kernel function.

SGWR builds on the GWR by allowing some variables to change from local to global, thus relaxing the GWR’s requirements for bandwidth limitations [40,61]. Further, to determine whether a variable is global or local by some algorithm:

(7) $$y_{i}=\beta_{j}\left(u_{j},v_{j}\right)+\sum_{k}\beta_{k}\left(u_{i},v_{i}\right)x_{ik}+\varepsilon_{i},$$

where *j* and *i* represent the global variables and local variables, respectively.

MGWR is also an extension to GWR, which further relaxes the GWR restriction to allow different bandwidths for each independent variable [41], so that different independent variables have different scale effects on the dependent variable:

(8) $$y_{i}=\beta_{0}\left(u_{i},v_{i}\right)+\sum_{k}\beta_{bwk}\left(u_{i},v_{i}\right)x_{ik}+\varepsilon_{i},$$

where *bwk* in $\beta_{bwk}$ represents the optimum bandwidth for the *k*th indicator.

The coefficient of determination (R2), the global Moran’s I of the residuals, the corrected Akaike Information Criterion (AICc), t testing, and F-testing were adapted to compare the performances of global regression models versus the SGWR and MGWR model with respect to the goodness-of-fit and residual spatial autocorrelation [33,42,62]. In this paper, both the SGWR and global regression models were built using the open source platform GWR 4 and the MGWR model was built via corresponding software MGWR.

## 4. Results

### 4.1. The Spatial Patterns of the LST and Landscape Indicators in Different Types of UFZs

#### The Spatial Patterns of the LST in Different Types of UFZs

**Figure 4:**
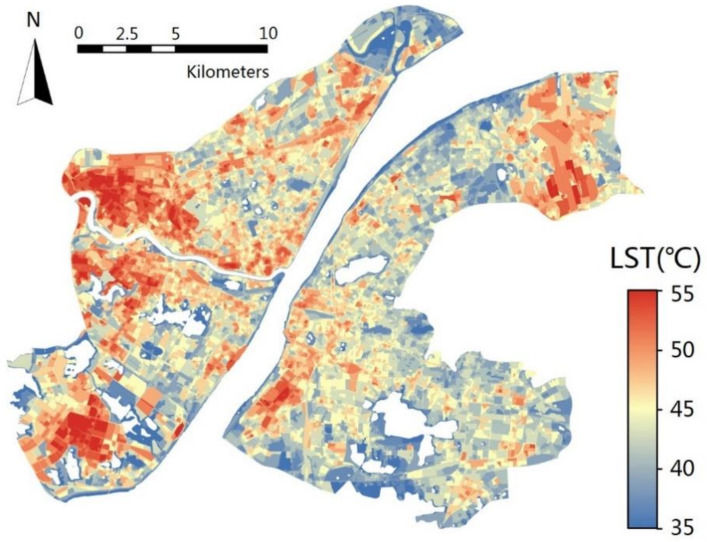
Average land surface temperature (LST) result in UFZs in Wuhan.

The LST distribution resampled in UFZs units in Wuhan is shown in Figure 4. The LST in different types of UFZs shows obvious spatial heterogeneity. According to statistics (as shown in Table 2), the maximum LST is concentrated in IM (48.88 °C) and LW (48.70 °C) and the minimum LST is concentrated in GS (43.80 °C) and W (41.61 °C). In particular, for built up areas, the lowest LST occurs in R (45.75 °C).

The LST within each UFZ also shows strong spatial heterogeneity due to the different locations and landscapes configuration. In fact, the difference between the maximum and minimum LST is almost 15~18° for all UFZs. 4.1.2. The Spatial Patterns of the Selected Indicators in different types of UFZs.

**Figure 5:**
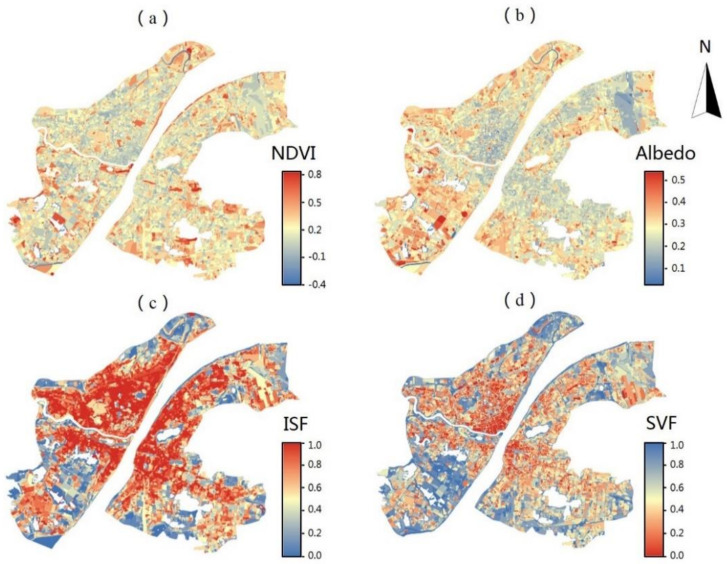
The spatial distributions of the selected indicators. (**a**) normalized difference vegetation index (NDVI); (**b**) Albedo; (**c**) impervious surface fraction (ISF); (**d**) sky view factor (SVF).

There are significant varieties in landscape compositions in different types of UFZs (as is shown in Figure 5, Table 3). For built up areas, CBF (0.37) and UV (0.37) have the highest BD and APS (0.20) has the lowest. The highest average BH is witnessed in R (23.78), almost twice as tall as the second-highest CBF (12.94), while IM (5.21) and LW (4.90) are the lowest. Meanwhile, R (5.72) is the UFZ with the highest BVD, followed by CBF (4.84), when UV (0.48), R (0.53), and CBF (0.54) are the UFZs with the lowest SVF. All built up areas have high ISF and the differences in ISF among different types of UFZs are small. In terms of NDVI, CBF (0.20) has the lowest NDVI, while in the built up area, APS (0.36) and R (0.31) have the highest NDVI. VL (0.22) has the highest albedo, showing high albedo for bare soil and hard pavement (mainly concrete), while R (0.16) has the lowest albedo, possibly related to the most pronounced street canyon effect in R.

### 4.2. Regression Results Analysis

#### 4.2.1. Performance Summary of the Global Regression Models

Comparing the global regression results for the entire city and the different type of UFZs (as is shown in Table 4), the selected seven indicators are more explanatory for the LST variability among UFZs and less explanatory for the LST variability within each UFZ. Comparing the two regression results, both are not highly descriptive for the LST variability within each UFZ. By contrast, RF regression has a higher goodness-of-fit than OLS, but the optimization is not obvious. In VL and RT, there is a low correlation between the selected indicators and LST in the global regression, so the spatial heterogeneity in the Landscape-LST relationship in these two types of UFZs will not be further discussed in subsequent studies.

#### 4.2.2. Performance Summary of the Random Forest Regression Models

**Figure 6:**
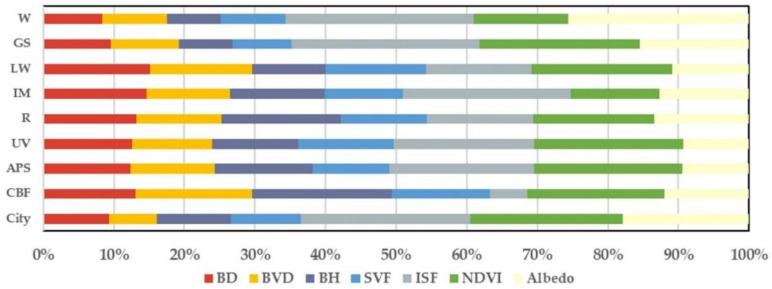
Comparison of the importance of landscape indicators regarding the LST in different UFZs by RF regression.

Comparing the RF regression results for the entire city and the different types of UFZs (as is shown in Figure 6), the four selected urban morphology indicators have higher explanatory power for the LST variability in the built up area, while the three urban landscape composition indicators have higher explanatory power for the LST variability in non-built-up areas. Among the different types of UFZs in built up areas, there is less variation in the correlation between selected indicators and LST. In particular, the most significant correlation between urban morphology indicators and LST was witnessed in CBF due to its high construction intensity. Specifically, in CBF, BH has the most significant impact on LST, while ISF has the lowest, however NDVI still indicates a significant correlation with LST. For landscape composition indicators, ISF and NDVI show the most significant impact on LST in most UFZs.

#### 4.2.3. Non-Stationarity of the Three Geographically Weighted Regression Models

Overall, both the SGWR and MGWR models are optimized based on the GWR model and the identification of the optimal bandwidth leads to an improvement in R^2^. The MGWR has a better goodness-of-fit, higher R^2^, lower AICC, and lower RSS than the SGWR. Comparing the OLS and RF regression, all three geographically weighted regression models show a higher goodness-of-fit (as is shown in Table 5).

**Figure 7:**
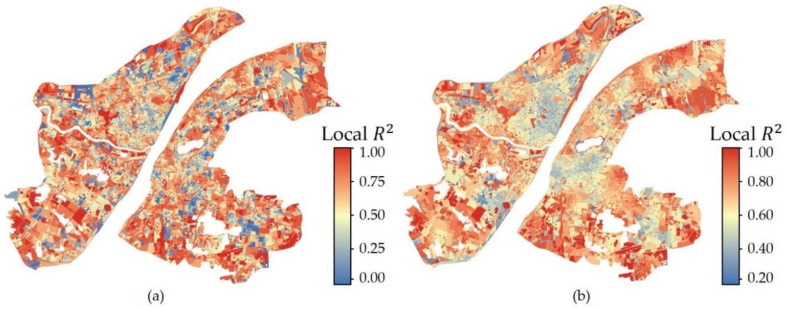
Local R^2^ for two GWR models (**a**) SGWR (**b**) MGWR.

The local R^2^ results (Figure 7) of the two GWR models show a clear spatial heterogeneity in the LST impact mechanism both among UFZs and within specific UFZ. Although each type of UFZ was regressed separately, the regression between LST and landscape indicators in the different types of UFZs have similar Local R^2^ distributions, with significant clustering of low and high values. Since the low local R^2^ values are clustered in areas with high population density and high construction intensity in the city center, one possible inference is that indicators reflecting anthropogenic heat such as population density and GDP per unit area need to be considered in future studies to explain the low local R^2^ in these areas.

**Figure 8:**
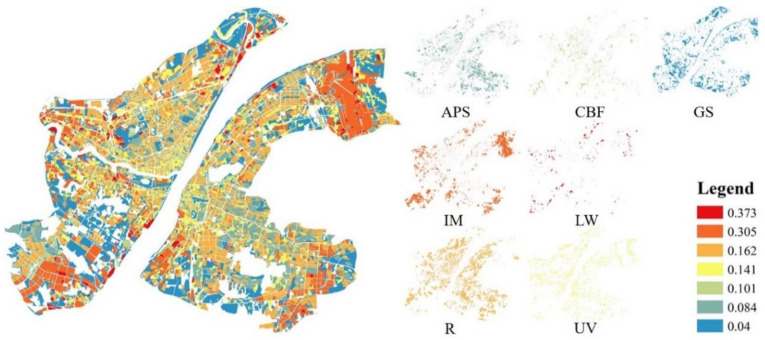
The slope coefficients of BD to LST by SGWR.

The slope coefficients of the selected indicators modeled by MGWR, SGWR are shown in Table 6. Both the OLS and RF regressions were modeled before the selected indicators participated in GWR models. Several selected indicators were not passing the p-test, for example, Albedo in UV; BH and SVF both in IM and LW; BVD in GS; and BD, BVD, BH, and SVF in W. Compared to the GWR, almost all of the selected landscape indicators interacted with LST in a global manner in almost all UFZs identified by the SGWR and MGWR (as is shown in Figure 8, for example, BD’s slope coefficients by SGWR). As is shown in Table 7, for SGWR, it generates an index to determine whether the contribution of the independent variable to the dependent variable is local or global, while for MGWR, a bandwidth of 30,000 m or more is considered a global indicator. At the same time, compared to the exhibited spatial heterogeneity of the selected indicators to LST among UFZs, the spatial heterogeneity is not evident within each UFZ. Since the local indicators impact LST in a global manner by GWR models, the slope coefficients of selected indicators did not differ much from the global regression. One of the largest differences compared to the global regression is that BD shows the strongest positive correlation in all six built up UFZs (CBF, ABS, R, UV, IM, LW), especially in IM and LW. Compared to the global regression in which the strongest positive correlation is ISF and the strongest negative correlation is NDVI, this shows the higher importance of urban morphological indicators in influencing LST as modeled by GWR. At the same time, both BVD and BH show a strong negative correlation when SVF is involved in the operation and this negative correlation is even stronger than NDVI in CBF. Meanwhile, due to the fact that the GWR model can perform a P-value test at every point, albedo does not pass the local P-test at many points in some UFZs. Besides, the slope coefficient of albedo is much lower in the geographically weighted regression models than in the global regression.

## 5. Discussion

This study investigated the spatial non-stationary associations between landscape indicators and LST through SGWR and MGWR. Compared to previous studies, which either utilized local regression models for the entire city’s LST mechanism exploration or focused on the diversified mechanisms among UFZs through global models, this study explored the geographically non-stationary nature of LST both among UFZs and within specific UFZs. A discussion of the results, applications, and limitations of this study is presented in the following.

### 5.1. Spatial Heterogeneity in Landscape-LST Relationship among UFZs and within Each-UFZ

Significant spatial heterogeneity in the Landscape-LST relationship is observed in different types of UFZs due to the impact of socioeconomic activities and related landscape characters. Diversified socioeconomic activities resulted in different anthropogenic heat, which has an important impact on urban LST [63]. In terms of anthropogenic heat, population density, traffic flow, industrial energy consumption, and building energy consumption are the most important components [64,65]. As a city with a relatively high proportion of heavy industry, industrial heat consumption is one of the main heat sources in Wuhan [66]. Further, the distribution of industrial land coincides with high-LST clustered areas. Energy consumption of commercial and residential buildings is generally considered to be an important source of urban anthropogenic heat [67]. Besides, commercial buildings during the day and residential buildings at night are often considered highly correlated with population concentrations due to the impact of work commuting [68]. So, CBF and UV have a higher LST, while R has a lower LST because the daytime LST images were selected. While road density and traffic flow have been found to have a strong positive correlation with LST in previous studies, in our study, RT itself was observed to have a lower LST than the surrounding built up area.

Both 2D and 3D building morphology show a significant correlation with LST variabilities in the built up area. However, these two types of indicators have variable importance in different types of UFZ. Both 2D and 3D building morphology indicators reflect the roughness of the urban surface and urban ventilation conditions, which conjointly regulate the urban surface thermal environment [69]. Nevertheless, these two types of indicators tend to influence urban LST in different ways. In particular, however, the 2D building morphology places more emphasis on building landscape, which changes the physical surface properties such as specific heat capacity and evapotranspiration efficiency [70], while the 3D building morphology can indicate the building’s shadow and incident solar radiation [30,71]. The correlation between 3D building morphology and LST was weak in IM and LW and neither BH nor SVF passed the P test. This is partly due to the significant correlation of industrial energy consumption with LST and the weak correlation of urban ventilation with LST and partly due to the small difference in industrial buildings’ BH and the weak correlation of differences in solar incident radiation and LST. In contrast, on account of the diverse building forms, high average building heights, and concentrated high-rise buildings in CBF and R, the 3D building morphology indicators showed a significant correlation to LST. BVD’s negative correlations to LST, BH’s negative correlations, and SVF’s negative correlations were observed when BVD, BH, and SVF were considered simultaneously. However, in regression with different combinations of independent variables, the positivity or negativity of this correlation is not invariable [34,72].

While landscape composition indicators such as ISF and NDVI have shown strong correlations in previous studies with LST differences between built up and non-built up areas, they tended to be less important than urban morphology indicators in built up areas. The transformation from natural surfaces to manmade surfaces, with the extensive use of reinforced concrete materials, has transformed the land surface from a high heat capacity environment to a low heat capacity environment [73]. Therefore, when exposed to solar radiation, the warming effect in built up areas is significantly higher than in non-built-up areas, which is the root cause of the extreme local warming effect in urban areas. At the same time, the transition from permeable to impervious surfaces attenuates the cooling effect of surface evapotranspiration on the land surface. However, the regression results showed that the positive correlation of ISF on LST and the negative correlation effect of NDVI on LST were not linear [74]. In fact, this correlation is weakened in the high construction intensity lands, especially in CBF. However, albedo, which was considered to be highly correlated with LST in previous studies, did not show a significant correlation in this study and did not even pass the P-test in OLS in several types of UFZs, although it still showed a high correlation in RF regression. This may be due to the complex impact mechanism of albedo on LST [75]. The use of light-colored building materials can reduce LST, showing a negative correlation between albedo and LST [76]. However, if solar incident radiation, material emissivity, and multidirectional reflectivity due to street canyon effect are considered, the complex impact mechanism of albedo on LST needs to be discussed more specifically, especially in the areas with high street canyon effects [75].

From our research, different landscape indicators tend to impact LST on a global manner within each UFZ. This may indicate that the various spatial indicators for LST act on the same spatial scale, while the same spatial indicator has the same correlation with the LST within each UFZ. This may be explained by the fact that the same type of UFZs not only have similar patterns of anthropogenic thermal effects, but also have similar landscape compositions and building form design criterion. Also, this could be a possible explanation for the significant spatial heterogeneity in the correlation between landscape indicators and LST at the intra-urban scale.

### 5.2. Planning Strategies Based on the UFZs Unit

This study validates the importance of UFZ as a fundamental LST research unit in urban planning applications. Urban planning at the UFZs scale is an important part of urban meso-scale planning. In China, regulatory detailed planning plays a corresponding function. Urban meso-scale planning is conducted in the urban zoning scale, city block scale, and building group scale. The importance of urban meso-scale planning can be seen in the following two ways. Urban meso-scale planning can translate urban climatology knowledge from macro-scale studies to planners in the form of diagrams and figures through urban zoning. Besides, it can provide a planning basis for building and landscape configurations in urban micro-scale planning. Meanwhile, the fact that landscape indicators impact on LST by similar mechanisms within each UFZ has important implications for urban planners as it means that planners can develop the same planning strategy for the same type of UFZ, simplifying the planning process.

Differential planning measures should be employed to mitigate the local warming effect in the different types of UFZs, referring to the significant spatial heterogeneity in the Landscape-LST relationship among UFZs. In detail, for CBF, APS, R, and UV, compared with the past low-rise, high-density, and high-rise high-density building development pattern, the high-rise low-density building pattern is more conducive to the improvement of urban ventilation conditions [77]. Besides, the high-rise low-density building pattern also means less solar incident radiation and more ecological landscape, thus lowering LST. Although, building density was one of the most important LST impact indicators both in this study and in previous studies. Building density cannot be declined indefinitely, taking into account the limits of socioeconomic activity. Building 3D morphology indicators play a more important role in mitigating local warming effects. Some measures, such as optimizing building form and street layout, could also be adopted. For IM and LW, due to the weak correlation between urban morphology and LSTs, as well as their homogeneous architectural patterns, we prefer not to propose corresponding planning measures in terms of building form. Besides, reducing building density and increasing green space are also limited in IM and LW. Adjusting the land configuration is considered another commonly used planning tool. IM and LW have a significant warming effect on the surrounding environment. Due to the disorderly planning, there has been a mixed layout of industrial and residential land uses in old cities, which is exacerbating the impact of industrial heat emission on residents’ health. According to our study, it is more appropriate for industrial land to be concentrated in industrial parks that are separated from the city center, especially for heavy industries that are more intrusive to the surrounding environment. Besides, the periphery of the industrial park could be set up with different widths of green belt according to the specification requirements, which can effectively reduce the heat exchange between the industrial park and the residential area. Although albedo did not show a strong correlation with LST in this study, the use of light-colored building materials is still considered a selectable planning measure in IM and LW as it has been considered an important LST impact indicator in previous studies.

### 5.3. Limitation and Future Work

There are several limitations worthy of further discussion. First of all, in contrast to traditional gridded-based research, UFZs-based research is able to avoid the influence of mixed-function in grid-based units. However, due to its inconsistent size, the most appropriate regression model should be both geographically weighted and area weighted. The conventional regression approach may amplify the interference of certain area extremums for regression as the spatial discordance of LST is more evident in small units. Secondly, the workflow proposed in this study can also be applied in other cities or at other times. Since the Landscape-LST relation may differ in variable climate zones, anthropogenic heat may also have different correlations in the diurnal and seasonal variations of LST. It is difficult to get the urban functional maps from the administration, so the method of identifying UFZs based on surface landscape characters can be explored in future studies. Thirdly, the LST images inversed by satellite sensors were found to be higher than the actual surface temperature during daytime under clear sky conditions due to the influence of urban 3D morphologies. Therefore, the interference of multi-directional radiation in urban remote sensing on surface temperature inversion and the correction of air temperature on surface temperature should be considered in future studies [78,79]. Above all, this study can be improved from two perspectives: (1) more indicators can be included in the regression to explain the under-discussed spatial heterogeneity of the LST influence mechanism, (2) and the workflow proposed in this study can also be applied in other cities or at other times.

## 6. Conclusions

In this study, UFZs were chosen as the basic research unit to discuss the spatial heterogeneity of different urban functions on the impact of LST. Global regression including OLS and RF regression were used to model the relationships between landscape indicators and LST and compare the importance of each landscape indicator, and spatial regression including SGWR and MGWR were applied to investigate the spatial heterogeneity in the Landscape-LST relationship among different types of UFZ and within each UFZ. The main findings could be summarized as follows: (1) Significant spatial heterogeneity in the Landscape-LST relationship was witnessed among UFZs, but the spatial heterogeneity is not obvious within specific UFZs. (2) The urban morphology shows a stronger correlation with LST differences within the built up area compared to the entire city, indicating the importance of ventilation and solar incident radiation for the urban thermal environment. (3) Within the same UFZ, each landscape indicators tend to impact LST on a global manner, which provides a perspective to investigate the drivers of the LST differences at the intra-urban scale. (4) Different planning strategies are proposed for different UFZs in terms of urban building form, landscape composition, and urban function organization to mitigate the local warming effect.

Overall, this study advances our understanding of human-environment interactions through the use of geospatial technologies and remote sensing for the study of urban climatological mechanisms. However, due to the absence of indicators characterizing population density and socioeconomic activity, the LST impact mechanism within each UFZ needs to be further discussed. In future studies, population density and socioeconomic indicators will be included in the discussion in more detail in order to investigate the urban LST impact mechanism and to better propose targeted urban planning strategies. Meanwhile, multi-spatial and multi-temporal data could be discussed further to discover more general conclusions.

## Figures and Tables

**Table 1:** Comprehensive information about the selected indicators.

Indicators	Description	Range
	**Landscape composition indicators retrieved from Landsat images**	
NDVI	Growth status, abundance, and coverage of vegetation, calculated as	[−1,1]
	(ρ(NIR) − ρ(Red))/(ρ(NIR) + ρ(Red))	
albedo	Overall reflectance in all directions	[0,1]
	**Landscape composition indicators retrieved from open-source datasets**	
ISF	Fraction of impervious surface	[0,1]
	**Morphology indicators retrieved from building survey data**	
BD	The total area of building divided by the pixel area	[0,1]
BH	The area averaged building height	[0,max]
BVD	A 3D indicator calculated as the building volume divided by the pixel area	[0,max]
SVF	The fraction of sky visibility at a given point	[0,1]

**Table 2:** The basic information of different UFZs.

UFZs Category	The Number of Zones	Average Area/m^2^	Area Percentage	Average LST/°C	**LST STD/**°C
Commercial and Business Facilities (CBF)	2201	10,304	4.45%	47.76	2.13
Administration and Public Services (APS)	3555	17,796	12.43%	46.34	2.04
Urban Village (UV)	2557	17,949	9.02%	47.50	2.13
Residential (R)	3920	25,016	19.26%	45.75	1.88
Vacant Land (VL)	1130	21,689	4.81%	46.89	2.04
Industrial and Manufacturing (IM)	2278	31,116	13.92%	48.88	2.52
Logistics and Warehouse (LW)	353	26,240	1.82%	48.70	2.33
Road and Transportation (RT)	4223	12,989	10.78%	46.70	2.25
Green Space (GS)	2376	39,120	18.26%	43.80	2.53
Wetland (W)	704	37,959	5.25%	41.61	2.82

**Table 3:** Indicators statistics in different UFZs.

Function		CBF	APS	UV	R	VL	IM	LW	RT	GS	W
BD/m^2^	mean	0.37	0.20	0.37	0.26	0.02	0.31	0.24	0.06	0.02	0.01
	std	0.17	0.09	0.14	0.05	0.02	0.15	0.17	0.07	0.02	0.01
BVD/m^3^	mean	4.84	2.30	3.37	5.72	0.19	1.62	1.19	0.59	0.14	0.05
	std	3.64	1.42	1.66	1.33	0.28	0.89	0.92	0.77	0.17	0.06
BH/m	mean	12.94	11.37	9.20	23.78	6.01	5.21	4.90	7.27	6.67	5.77
	std	9.31	4.66	3.20	5.89	5.58	2.52	2.32	5.89	4.01	3.36
SVF	mean	0.54	0.70	0.48	0.53	0.96	0.64	0.71	0.95	0.96	0.98
	std	0.18	0.13	0.18	0.08	0.04	0.16	0.19	0.12	0.03	0.02
ISF	mean	0.84	0.70	0.81	0.81	0.59	0.75	0.80	0.82	0.33	0.26
	std	0.17	0.23	0.17	0.10	0.29	0.19	0.18	0.24	0.23	0.20
NDVI	mean	0.20	0.36	0.26	0.31	0.23	0.25	0.25	0.29	0.48	0.37
	std	0.09	0.09	0.09	0.04	0.08	0.08	0.09	0.11	0.11	0.20
Albedo	mean	0.20	0.18	0.17	0.16	0.22	0.20	0.20	0.18	0.19	0.16
	std	0.04	0.02	0.02	0.01	0.02	0.05	0.04	0.03	0.02	0.05

**Table 4:** An evaluation summary of the goodness-of-fit (R^2^) of ordinary least squares (OLS), random forest regression (RF).

	City	CBF	APS	UV	R	VL	IM	LW	RST	GS	W
OLS	0.40	0.26	0.31	0.41	0.33	0.06	0.31	0.31	0.21	0.33	0.29
RF	0.45	0.28	0.32	0.42	0.35	0.05	0.34	0.28	0.20	0.38	0.37

**Table 5:** An evaluation summary of the performance of ordinary least squares (GWR), geographically weighted regression (SGWR), and multi-scale geographically weighted regression (MGWR).

		CBF	APS	UV	R	IM	LW	GS	W
GWR	R^2^	0.65	0.65	0.71	0.71	0.76	0.54	0.67	0.67
	AICC	4322.82	6267.77	4450.93	7155.12	3694.50	618.97	5096.00	1405.31
	RSS	571.37	858.97	569.72	1058.59	389.79	92.02	625.58	161.62
SGWR	R^2^	0.77	0.73	0.82	0.76	0.79	0.58	0.71	0.66
	AICC	4047.80	5589.98	3949.39	6771.26	3181.71	550.22	4741.73	1321.99
	RSS	502.37	595.65	378.35	796.63	372.44	89.31	513.78	152.71
MGWR	R^2^	0.81	0.81	0.84	0.82	0.85	0.76	0.78	0.76
	AICC	3582.77	5244.34	3597.96	9541.57	3041.07	545.31	3853.85	1269.37
	RSS	303.14	475.57	307.35	526.68	248.89	71.35	381.26	130.22

**Table 6:** The slope coefficients of the indicator to LST by MGWR, SGWR.

		BD	BVD	BH	SVF	ISF	NDVI	Albedo
CBF	MGWR	0.097	−0.112	−0.129	−0.092	0.069	−0.15	0.002
	SGWR	0.101	−0.111	−0.141	−0.09	0.071	−0.161	0.003
APS	MGWR	0.072	−0.104	−0.066	−0.032	0.157	−0.203	−0.011
	SGWR	0.084	−0.124	−0.068	−0.05	0.192	−0.214	−0.01
UV	MGWR	0.14	−0.114	−0.059	−0.101	0.125	−0.202	*p* < 0.05
	SGWR	0.141	−0.121	−0.051	−0.107	0.143	−0.201	
R	MGWR	0.16	−0.14	−0.11	−0.069	0.123	−0.181	0.051
	SGWR	0.162	−0.147	−0.124	−0.083	0.134	−0.186	0.049
IM	MGWR	0.305	−0.189	*p* < 0.05	*p* < 0.05	0.153	−0.107	−0.018
	SGWR	0.305	−0.171			0.164	−0.119	−0.025
LW	MGWR	0.339	−0.146	*p* < 0.05	*p* < 0.05	0.189	−0.276	−0.06
	SGWR	0.373	−0.191			0.225	−0.287	−0.04
GS	MGWR	0.03	*p* < 0.05	−0.043	−0.067	0.282	−0.267	0.174
	SGWR	0.04		−0.053	−0.06	0.315	−0.261	0.168
W	MGWR	*p* < 0.05	*p* < 0.05	*p* < 0.05	*p* < 0.05	0.3	−0.058	0.306
	SGWR					0.375	−0.055	0.348

**Table 7:** Optimal bandwidths identified by GWR, SGWR, and MGWR.

		CBF	APS	UV	R	IM	LW	GS	W
Intercept	GWR	996.64	847.18	1024.88	997.24	847.72	3156.730	1042.39	1277.92
	SGWR	558.34	341.74	369.73	567.27	395.32	1711	682.05	891.66
	MGWR	221.77	228.33	259.64	201.61	274.07	1277.31	340.66	631.03
BD	GWR	996.64	847.18	1024.88	997.24	847.72	3156.730	1042.39	*p* > 0.05
	SGWR	global	global	global	global	global	global	global	
	MGWR	76,258.54	77,360.32	74,474.13	77,866.42	2402.03	34,814.55	79,683.88	
BVD	GWR	996.64	847.18	1024.88	997.24	847.72	3156.730	1042.39	*p* > 0.05
	SGWR	global	global	global	567.27	global	global	global	
	MGWR	8090.44	77,360.32	74,474.13	77,866.42	3150.17	70,975.55	2237.86	
BH	GWR	996.64	847.18	1024.88	997.24	*p* > 0.05	*p* > 0.05	1042.39	*p* > 0.05
	SGWR	global	global	global	global			global	
	MGWR	76,258.54	77,360.32	74,474.13	7833.11			79,683.88	
SVF	GWR	996.64	847.18	1024.88	997.24	*p* > 0.05	*p* > 0.05	1042.39	*p* > 0.05
	SGWR	global	global	global	global			global	
	MGWR	76,258.54	77,360.32	5292.86	13,689.35			79,683.88	
ISF	GWR	996.64	847.18	1024.88	997.24	847.72	3156.730	1042.39	1277.92
	SGWR	global	341.74	369.73	567.27	global	global	682.05	global
	MGWR	76,258.54	5085.64	2318.58	77,866.42	78,630.13	3516.16	807.47	2301.5
NDVI	GWR	996.64	847.18	1024.88	997.24	847.72	3156.730	1042.39	1277.92
	SGWR	558.34	global	global	567.27	global	global	682.05	global
	MGWR	76,258.54	77,360.32	7796.52	2129.01	5596.41	7144.02	1310.45	78,682.86
albedo	GWR	996.64	847.18	*p* > 0.05	997.24	847.72	3156.730	1042.39	1277.92
	SGWR	558.34	global		567.27	global	global	682.05	891.66
	MGWR	76,258.54	77,360.32		77,866.42	5116.48	70,975.55	1196.87	2214.13

## Abbreviations

LCZ	Local climate zones
UFZ	urban functional zone
LST	land surface temperature
OLS	ordinary least square regression
RF	random forest regression
SGWR	semi-parametric geographically weighted regression
MGWR	multiscale geographically weighted regression
2D	two-dimensional
BD	building density
3D	three-dimensional
BH	building height
BVD	building volume density
SVF	sky view factor
NDVI	normalized difference vegetation index
ISF	impervious surface fraction
R	Residential
UV	Urban village
APS	Administration and Public Services
CBF	Commercial and Business Facilities
IM	Industrial and Manufacturing
LW	Logistics and Warehouse
GWR	geographically weighted regression
GS	Green Space
RT	Road and Transportation
VL	Vacant Land
W	Wetland
